# Prevalence and risk factors for overweight horses at premises in Sweden assessed using official animal welfare control data

**DOI:** 10.1186/s13028-016-0242-3

**Published:** 2016-10-20

**Authors:** Peta L. Hitchens, Jan Hultgren, Jenny Frössling, Ulf Emanuelson, Linda J. Keeling

**Affiliations:** 1 Department of Animal Environment and Health, Swedish University of Agricultural Sciences (SLU), P.O. Box 7068, 750 07 Uppsala, Sweden; 2 Department of Animal Environment and Health, Swedish University of Agricultural Sciences (SLU), P.O. Box 234, 532 23 Skara, Sweden; 3 National Veterinary Institute, 751 89 Uppsala, Sweden; 4 Department of Clinical Sciences, Swedish University of Agricultural Sciences (SLU), P.O. Box 7054, 750 07 Uppsala, Sweden; 5 Equine Centre, Faculty of Veterinary and Agricultural Sciences, University of Melbourne, 250 Princes Hwy, Werribee, Victoria 3030 Australia

**Keywords:** Epidemiology, Legislation, Compliance, Logistic regression, Horse, Body condition, Welfare, Welfare assessment, Equine, Obesity

## Abstract

**Background:**

There are Swedish animal welfare regulations concerning the body condition of horses and general advice on keeping horses including that horses should be fed so that they do not become over- or underweight relative to their use. Compliance is assessed by official animal welfare inspectors. The objective of this study was to determine whether the national animal welfare control database could be used to estimate the prevalence and risk factors for overweight horses in Sweden. The official animal welfare control checklist for horses contains 45 checkpoints (CP) of which CP-8 pertains to the acceptability of the horses’ body condition including whether they were under- or overweight. Prevalence of non-compliance with CP-8, with 95 % confidence intervals (CI), were calculated for the years 2010–2013. Associations between risk factors and non-compliance for overweight body condition were estimated using logistic regression and expressed as odds ratios (OR) with 95 % CIs.

**Results:**

Of 7870 premises with registered horses that were inspected against CP-8, a total of 63 premises had non-compliant inspections due to overweight horses (0.80 %; CI 0.62, 1.02 %). In multivariable analyses, premises that were non-compliant with requirements for the care of sick or injured horses (OR 3.52; CI 1.51, 8.22) or with the requirements for feeding a balanced high-quality diet (OR 5.15; CI 2.49, 10.67) had greater odds of having overweight horses. Premises that also kept other species for meat production were more likely to have overweight horses (OR 2.12; CI 1.18, 3.81) whereas professional horse establishments were less likely (OR 0.09; 0.01, 0.64). Overweight horses were more likely in summer compared to winter (OR 2.18; CI 1.02, 4.70). Premises in regions of Sweden with more horses in relation to the human population were less likely to have overweight horses (OR 0.97; CI 0.95, 1.00).

**Conclusions:**

Official animal welfare control data may be used to monitor the premises prevalence of overweight horses in Sweden. Strategies to reduce the prevalence of overweight horses should focus on education about equine care and nutrition, especially summer grazing.

**Electronic supplementary material:**

The online version of this article (doi:10.1186/s13028-016-0242-3) contains supplementary material, which is available to authorized users.

## Findings

To ensure compliance with Swedish animal welfare legislation and with European Union (EU) regulation (EC) 882/2004, there is an animal welfare control system in Sweden whereby official inspectors visit horse premises. One of the legislative requirements is that horses should be fed so that they do not become over- or underweight relative to their use [1, 2].

Equine obesity is a welfare issue that has been associated with laminitis, insulin resistance and other metabolic health problems [3]. Investigations of the prevalence of equine obesity have been inconsistent and, in studies conducted this decade, this has resulted in ranges from 19 to 45 % [4–9]. These studies differed in body condition scoring or use of owner reported information [7, 8, 10], and were conducted at riding clubs or equestrian organisations [4, 6], or in sub-populations of competition [9] or mature horses [5]. Seasonal differences [4], breed or type (e.g. draught-type, cob-type and native) [4, 9, 10], and how the horses were used (pleasure riding and non-ridden horses compared to competition horses) [10] have been identified as factors associated with increased prevalence of equine obesity.

The objective of this study was to determine whether the Swedish official animal welfare control database could be used to estimate the prevalence and risk factors for premises being non-compliant for body condition because at least one horse was overweight or obese. This study is part of a larger study assessing animal welfare problems using data from official animal welfare control (Hitchens et al. submitted).

Complete data from official animal welfare control in all 21 counties of Sweden from 1 January 2010 to 31 December 2013 were provided by the Swedish Board of Agriculture (JV). The dataset and methods have been detailed previously (Hitchens et al. submitted).

One of the control checklists refers to compliance with legislative requirements for the keeping of horses [1, 2]. The horse checklist contains 45 checkpoints (CP) relating to animal-, resource- and management-based measures. Data from CP-8 pertains to whether the horses’ body condition is acceptable. The control result was either not applicable, no control carried out, compliant, or non-compliant. Inspections on the horse checklist were conducted by official animal welfare inspectors employed by the county administrative boards (n = 323 inspectors). Inspectors were educated about body condition scores from the Carroll and Huntington [11] five-point scaling system. Inspectors assessed non-compliance with CP-8 as at least one horse having a body condition score at the most severe point (i.e. one for underweight, or five for overweight), and could use their discretion based on other factors or extenuating circumstances if any horses were assessed as a two or four; but comments in the database only specify whether the horse(s) are under or overweight. Outcome indicators of body condition in this study were defined as ‘overweight’—coded as non-compliant with CP-8 because of at least one overweight horse on the premises (1), all others were compliant with CP-8 (0). Inspections that did not have a reason for non-compliance (n = 34) and those that were conducted at events (e.g. exhibition or competition) were excluded from analysis (n = 65). Since complete information on the number of horses at each premises was not available, only the premises prevalence of non-compliance, i.e. at least one over- or underweight horse, with CP-8 could be calculated; 95 % confidence intervals (CI) were estimated using the exact binomial distribution.

Models were generated at the inspection level because study factors changed between inspections. Univariable logistic regression was used to associate occurrence of overweight horses with factors related to the geographical region and type of premises (Additional file 1: Table S1), factors related to the inspection (Additional file 2: Table S2), and resource- and management-based measures (Additional file 3: Table S3). The predictors from univariable analyses with p < 0.2 were entered into a multivariable model and retained in the model if they were statistically significant (p ≤ 0.05) using backward stepwise elimination. Two-way interactions between each of the main effects retained in multivariable model were assessed, but none were significant. Odds ratios (OR) and their 95 % CIs, with standard errors adjusted for clustering on premises, are presented. Linearity for continuous variables was assessed by generating Box-Tidwell power transformations. Model diagnostics included the Hosmer–Lemeshow’s goodness-of-fit test, link test, and examination of tolerance (>0.1) and variance inflation factor (VIF < 10). Statistical analyses were conducted using Stata, version 13.1 (StataCorp, College Station, Texas, USA).

Eighty-six percent of the horse premises inspections (n = 11,397, from a total of 13,321) included inspections related to CP-8 on the horse checklist. Multiple inspections were conducted on some premises, resulting in 7870 different premises with horses being inspected against CP-8. Of these, a total of 63 premises had non-compliant inspections against CP-8 due to overweight horses; 55 of these premises had only one non-compliant inspection, six were still non-compliant on the second inspection, one on the third, and one on the fourth inspection. The premises prevalence of having overweight horses was lower than for underweight horses (Table 1).

**Table 1:** Inspection results for body condition

Compliance	Number of inspections	Number of premises	Premises-level prevalence (95 % CIs)
Compliant	10071	6937	–
Non-compliant, overweight	74	63	0.80 (0.62–1.02)
Non-compliant, underweight	1227	861	10.96 (10.27–11.67)
Non-compliant, CP-8	1326	933	11.86 (11.15–12.59)
Total inspections	11397	7870	–

Percentage of inspections and premises non-compliant with checkpoint 8, where the inspector reported at least one horse as over- or underweight

Nine inspections had both under and overweight horses recorded; 1726 (13.0 %) visits did not inspect horses and 133 (1.0 %) visits were not applicable for checkpoint 8. Reasons for non-compliance were missing for 34 inspections and 11 premises, thus these have been excluded from the total denominator in calculations of prevalence for reasons of being under or overweight

In multivariable analysis, premises that were non-compliant with requirements for the care of sick or injured horses (CP-12), and premises non-compliant with requirements for feeding a balanced high-quality diet (CP-29) had greater odds of having overweight horses (Table 2). Overweight horses were more likely to be observed in summer compared to winter. Premises that kept other species for meat production, that were not professional horse establishments, and/or were located in regions of Sweden that had fewer horses in relation to the human population (i.e. urban areas) were also all more likely to have overweight horses. There were no significant interactions and no significant effect of year.

**Table 2:** Risk factors associated with overweight horses

Variable	Overweight or obese			
	Compliant	Non-compliant	OR (95 % CI)	*P* value
Care of sick animals (CP-12)				
Compliant	6646	19	Ref	
Non-compliant	711	23	3.52 (1.51–8.22)	0.004
Not applicable/not inspected	3932	32	1.63 (0.84–3.19)	0.149
Quality feed (CP-29)				
Compliant	8485	29	Ref	
Non-compliant	631	23	5.15 (2.49–10.67)	<0.001
Not applicable/not inspected	2173	22	1.77 (0.93–3.37)	0.079
Season				
Winter	2887	11	Ref	
Spring	3144	21	1.51 (0.69–3.31)	0.301
Summer	2262	23	2.18 (1.02–4.70)	0.045
Autumn	2996	19	1.68 (0.79–3.57)	0.180
Horses per human pop^n^ (/1000)^a^	–	–	0.97 (0.95–1.00)	0.030
Professional establishment^b^				
No	8558	73	Ref	
Yes	2731	1	0.09 (0.01–0.64)	0.016
Meat production				
No	9074	52	Ref	
Yes	2215	22	2.12 (1.18–3.81)	0.012

Multivariable analysis of associations between risk factors and non-compliance with checkpoint 8 (body condition) where the inspector reported at least one horse as overweight, adjusted for clustering on premises (n = 10,796). Raw data for frequencies of compliant and non-compliant inspections are presented

^a^Number of compliant and non-compliant inspections are not reported for continuous variables

^b^Holds a permit under 16 § of the Swedish Animal Welfare Act, for “an operating permit required by any person who, on a professional basis or on a substantial scale: 1. keeps, breeds, supplies or sells pet animals or receives pet animals for boarding or feeding; 2. keeps, breeds, supplies or sells horses or receives horses for boarding or feeding or uses horses in a riding school business; or 3. breeds fur animals.”

This is the first study to report the prevalence of overweight horses at national level using routinely collected official animal welfare control data. Similar to the 2005 NAHMS study [12], we also report premises prevalence, where other studies report prevalence at the individual horse-level. The premises prevalence reported in the NAHMS study [12] is higher (3.4 %) than ours (<1 %), but these were owner-reported assessments and thus not directly comparable. In the current study, observation of underweight horses was more common. The study identified several risk factors that were associated with a horse being so overweight that it was considered to be non-compliant with the Swedish animal welfare legislation, but due to the small number of these non-compliant inspections, the results should be considered with caution.

Poor management, such as inadequate feeding of a nutritionally balanced diet and care of sick and injured horses, was associated with overweight. It has previously been reported for horses in Great Britain, that being turned out for a greater amount of time per week, with no to low intensity exercise, and/or not feeding supplementary feed (e.g. concentrates) was associated with increased odds of owner-reported obesity [10]. Conversely, in another English study, supplementary feeding during winter was associated with increased odds of obesity, though these findings did not remain in multivariable analysis [4]. Giles et al. [4] found that horses that sustained an injury between winter and summer had greater odds of obesity compared to those without injury. We did not have direct information on injury to horses, however we did find that premises that were ill equipped to care for such sick or injured horses appropriately had higher odds of having overweight horses. Our results, in combination with previous studies, lead to the suggestion that strategies aimed at reducing the occurrence of overweight horses should focus on improving management-related factors for example by increasing education on equine care and nutrition.

There was an effect of season, with overweight horses more likely to be observed in summer when compared to winter. Giles et al. [4] similarly reported an increased prevalence of obesity in summer compared to winter by comprehensively assessing body condition scores of the same horse population at the two seasons. Increasing body condition score has also been shown to be associated with the date of examination in a summer study, suggesting that body condition scores increase as the summer progresses [13]. Our results therefore support the recommendation that horses prone to gaining excess weight should be monitored closely and grazing on summer pasture restricted if necessary.

Professional horse establishments, defined as operating an equine business on a substantial scale, had fewer problems with overweight horses. One possible reason for this may be that professional horse establishments, for example riding schools and studs, are likely to employ people knowledgeable about horse-keeping, and may differ in their management and exercise practices. On the other hand, premises that were involved in beef, pork or other meat production, were more likely to have overweight horses, perhaps implying that people in the business of fattening other production animals for meat production may inadvertently over-feed their horses. Premises in more rural regions had fewer problems with overweight horses. These results imply that characterising the number and type of animal-related activities on premises may help identify premises with a high risk of having overweight horses, so facilitating the efficient targeting of advice.

We have demonstrated the potential of epidemiological analyses of a national animal welfare database, but there are also limitations. It is difficult to compare studies because the definition of an overweight or obese horse varies [4], and because prevalence here is recorded at the premises-level, not at the horse-level. Reports of non-compliance with the checkpoint related to adequate body condition did not have information on the body condition score of the horse, and were missing in some instances. As such, improvements to data collection including recording of body condition score assessments, number of horses affected and number of horses assessed for this particular checkpoint are warranted.

Official animal welfare control data may be used to monitor the occurrence and risk factors for overweight horses in Sweden; however improvements to the database are needed. Strategies to reduce the prevalence of overweight horses should focus on education about equine care and nutrition, especially summer grazing.

## Additional files

**Additional file 1: Table S1.** Premises characteristics associated with overweight horses.

**Additional file 2: Table S2.** Inspection characteristics associated with overweight horses.

**Additional file 3: Table S3.** Resource- and management-based characteristics associated with overweight horses.
